# Over-the-Counter (OTC) Hearing Aid Availability across the Spectrum of Human Skin Colors

**DOI:** 10.3390/audiolres14020026

**Published:** 2024-03-12

**Authors:** Shade Avery Kirjava, Sam Jones Faulkner

**Affiliations:** 1 Department of Health, Society, and Behavior, University of California, Irvine, CA 92697, USA; 2 Program in Inclusive Design, OCAD University, Toronto, ON M5T 1W1, Canada; samfaulkner@ocadu.ca

**Keywords:** over-the-counter hearing aids, race factors, hearing aids

## Abstract

Background: Over-the-counter (OTC) hearing aids were recently approved for sale in the United States. Research has shown that consumers prefer hearing devices that match their skin color because these devices are less noticeable. Colorism is discrimination against individuals with relatively darker skin that manifests in “skin-color” product offerings as products being offered primarily in relatively lighter colors. Methods: This study compared images of U.S. Food and Drug Administration (FDA)-registered over-the-counter hearing aids to a range of human skin colors. Results: Most over-the-counter hearing aids are only offered in relatively lighter beige colors. Few over-the-counter hearing aids are available in darker skin colors. Conclusions: These findings may represent structural bias, preventing equitable access to darker skin-color OTC hearing aids for individuals with darker skin.

## 1. Introduction

Hearing loss is a critical public health concern. It affects about 1.5 billion people globally and costs about $980 billion USD in lost productivity alone annually [1]. The most common types of hearing loss, age-related and noise-induced hearing loss, are irreversible and typically treated with hearing aids prescribed by licensed hearing healthcare providers. In 2022, the FDA approved the sale of over-the-counter (OTC) hearing aids in the United States to reduce barriers in hearing healthcare treatment access [2]. However, unequitable OTC hearing aid device offerings threaten the potential of OTC hearing aids to reduce hearing health inequity. OTC hearing aids are non-prescription hearing aids designed as a more affordable alternative to prescription hearing aids, which are not covered by traditional Medicare and can cost upwards of $5000 [3,4,5]. The social epidemiologic framework of factors driving hearing health outcomes introduced by Nieman et al. positions OTC hearing aid use as an important factor in reducing hearing health inequity [3,4,5,6]. The model by Nieman et al. also suggests that racism and stigma about wearing hearing aids are key structural factors in understanding rates of hearing aid use.

Structural racism in the form of colorism has not been evaluated in the United States OTC hearing aid market. If present, this colorism may potentially limit OTC hearing aid uptake for individuals with relatively darker skin. As OTC hearing aids are an emerging market in the United States, there is room for great improvement in product selection, as hopefully the awareness of colorism with these devices will change future colorway options. Colorism is defined as the additional marginalization of BIPOC (Black, indigenous, and people of color) people with darker skin tones compared to peers with lighter skin tones and the valuing of features akin to euro-centric whiteness causing products in “skin colors” to be offered primarily or solely in relatively lighter colors [7]. Many industries have been reckoning with colorism in their product offerings in the aftermath of the 2020 George Floyd protests [8]. Brands such as Band-aid [9] and Crayola [10] have since launched or re-launched products that give more comprehensive skin tone options in an attempt to develop more equitable product portfolios. While the changes that happened in 2020 across the marketing and product development spheres were the beginning of a broader shift across industries, these more diverse skin-color product offerings were not adopted in all industries.

Hearing aids are one of the many industries that must consider their skin-color product offerings. Traditionally, a hearing aid was designed to blend in with the user’s skin or hair color. This design choice was made primarily due to the stigma associated with individuals wearing hearing aids being viewed as elderly or disabled. While the stigma has slowly become less prevalent in recent years, most users still prefer more discreet devices [11,12,13,14]. Having the option for hearing aid shells that blend into the user’s skin or hair color is an important consideration for hearing aid companies to consider when developing product colorways.

This study examines if OTC hearing aids in skin colors across the spectrum of typical human skin are available for purchase in the United States. It was predicted that there would be more OTC hearing aid offerings in relatively lighter skin colors compared to relatively darker skin colors. The goal of this study is not to perpetuate the stigma that hearing aids should be discreet, invisible, and hidden by means of blending the devices into a user’s hair or skin color. Instead, the goal is to highlight the inequitable device offerings that then limit a user’s ability to decide for themselves what they feel most comfortable with.

### 1.1. Colorism

Colorism is a phenomenon that has long been intertwined with the United States’ racist history [7]. The issue of preferring lighter skin, especially in marketing, has become more frequently challenged in the wake of the Black Lives Matter protests in 2020 [15]. However, despite the backlash companies like Heineken, Crayola, and Band-Aid have faced, colorism is still prevalent in many industries globally [15]. While this paper will specifically be considering the consequences of colorism in OTC hearing aids, the impact of the effect must be understood to contextualize these consequences, especially as colorism permeates many different aspects of everyday life. The effects of colorism are so far-reaching that it has even been documented that children in South Africa are more likely to use lighter crayons when drawing self-portraits despite them not matching their own darker skin tones [16]. With society often promoting and valuing relatively lighter euro-centric skin, large communities of people can feel unrepresented and can begin to internalize the idea that dark skin is not “pretty” or “worthy” of appreciation [17]. Not only are the effects of colorism attributed to negative self-perception, but there is evidence that people with darker skin tones are more likely to be fired and live in lower socio-economic conditions due to this racial bias [18].

### 1.2. Healthcare Inequality and Racism

After understanding the impacts of colorism, it is important to build on its specific relationship to healthcare. BIPOC people are often more apprehensive about accessing medical care due to a history of prejudice and discrimination from the healthcare system (that continues in many places to this day) [13]. In hearing care, racial disparities persist and are subject to the same issues as the rest of the healthcare system. For example, white people are more likely to report regular hearing aid use despite Black people having had more recent hearing tests [19]. This is partly due to two race-related factors, one being that white people are more likely to have higher socio-economic status than Black people living in the United States, and hearing tests are covered by Medicare but hearing aids are not [19,20]. Since Black people are more likely to be less affluent than their white peers, their lower hearing aid use rates can be related back to the combination of the high cost of the devices along with the lack of adequate insurance coverage [6].

### 1.3. Social Perceptions of Hearing Aids

It is now evident that BIPOC people are less likely to have access to hearing aids, so one must next consider perceptions of hearing aids themselves and how that may also influence device uptake rates. Hearing aids are often associated with negative aspects of aging, so much so that the, “Hearing Aid Effect” was coined to describe the stigma that people wearing hearing aids experience [14,21,22]. This stigma can lead to not only a reduced uptake in hearing aids but an increase in social isolation, loneliness, and negative self-perception [11,21]. Notably, a study on perceptions of hearing aids indicated that the “Hearing Aid Effect” is present for brightly colored hearing devices, but not ones matching skin tone [23]. If hearing devices are not available in the participant’s skin tone, the Hearing Aid Effect stigma becomes more impactful. This means that BIPOC people who are trying to access hearing aids but are worried about the consequences associated with the device stigma do not have the same ability to mitigate those concerns as white hearing aid users do.

### 1.4. Consequences of Unmanaged Hearing Loss

Managing hearing loss can look different depending on the kind of loss and the resources the individual has access to, but typically, hearing loss is addressed with the use of hearing aids [24]. So, if an individual cannot afford hearing aids or is too ashamed due to the stigma of wearing them, how will that impact the person’s life moving forward? Hearing loss has been linked to an increased risk of dementia and general cognitive decline, at least partly due to the additional processing required to participate in a conversation [24,25,26]. People who do not have the financial or social security required for hearing aids are at a greater disadvantage when trying to address their hearing loss. Since it has already been established that BIPOC people are less likely to be able to afford care and face additional social scrutiny, a conclusion can be drawn that BIPOC communities with hearing loss are at a greater risk of experiencing cognitive decline.

### 1.5. Over-the-Counter Hearing Aids

Next, it is important to consider what is being done in the hearing health industry to attempt to reduce some of the barriers like prohibitively high device costs and increased social stigma. When the FDA authorized the sale of OTC devices, they specifically cited both the high cost and the social stigma associated with hearing aids as reasons why OTCs needed to be introduced to the market [2]. These devices are designed to disrupt the high device cost by offering self-testing and fitting, which eliminates the need for going to an audiologist, which, as established above, can be a barrier in and of itself for BIPOC people needing healthcare. While OTCs are not meant to replace the field of audiology, given that the average delay between when someone realizes they have a hearing loss and them attempting to manage it is nine years, they are a good entry point to close that gap and mitigate the effects that can lead to dementia [27]. The same study on the time between hearing aid candidacy and adoption even notes that BIPOC folks tend to have even longer delays than white folks [27]. Given that communicating aurally with unmanaged hearing loss causes increased cognitive effort, which can quickly lead to cognitive decline, it is crucial for the nine-year gap to be minimized as much as possible.

### 1.6. Knowledge Gap

From reviewing the literature on the intersection of colorism and hearing health, it has become evident to the authors that hearing healthcare services, including access to hearing aids, are not equitably available to all populations, with reduced access to hearing healthcare for some historically marginalized populations, including BIPOC individuals. There is also a lack of information on hearing aid use and acceptability for BIPOC people in the United States. While there exist a few more modern papers, it is challenging to obtain data due to the lack of trust between BIPOC patients and medical personnel, especially after tragedies like the Tuskegee incident [28]. Finally, since OTCs are relatively new at the time of this study, there are relatively sparse data on their usage compared to other medical devices [2]. This research attempts to assess the availability of OTC hearing aids across the range of possible human skin colors.

## 2. Materials and Methods

**Figure 1:**
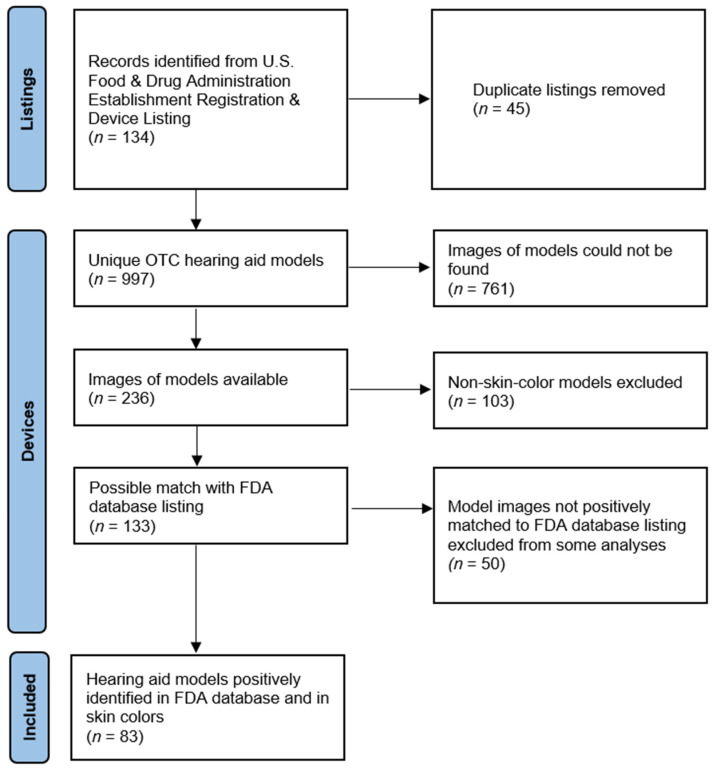
Data collection process.

For this study, the U.S. Food and Drug Administration Establishment Registration and Device Listing database was used to identify FDA-cleared over-the-counter hearing aids in August 2023, as shown in Figure 1 [29]. The database was searched for devices with product codes QUF, QUG, QUH, and QDD, which identify hearing aids for sale without a prescription [30]. All companies with devices registered with these product codes were extracted from the database in August 2023. Duplicates were defined as the same products being listed by several regional divisions of the same firm (e.g., a single firm registering a device separately with both the firm’s South African and Danish divisions). For products with duplicate entries, only a single product was included in the analyses to avoid over-representing duplicates in the analysis.

### 2.1. Data Collection

Google and Bing search engines were used in August and September 2023 to identify images for the hearing aid models listed in the FDA database. Search strings with various combinations of the company name and hearing aid model name or number were used to identify the company’s website or the online marketplace where the hearing aids were listed. These websites were used to identify and download images of each model hearing aid listed in the FDA database. Where a definite match between model name or number and image could not be made due to lack of a model name or model number listed in the FDA database, all potential device matches by that company were downloaded for analysis. Only behind-the-ear (BTE) models were included in the analysis, as in-the-ear (ITE) styles are often made to resemble commercially available Bluetooth headsets designed for phone calls, not to blend into the patient’s skin or attempt to make themselves less visible by having a smaller size, instead of matching an individual’s skin color.

### 2.2. Color Extraction

Where multiple images were available for a given hearing aid model, images that displayed the lateral side view of the device, that were real depictions of the device (as opposed to computer-generated renderings), that had frontal lighting, and that were not being worn by a person in the image were chosen. A single pixel was extracted from the central area of the lateral hearing aid casing for each device and was chosen to be representative of the color of the hearing aid casing. The extracted color was not chosen from areas with text, logos, or a casing seam. The single HEX color value for each device was converted to a CIELAB color space value, an international standard for defining the color of an object. Where a single hearing aid model was offered in several colors, the process was repeated for each model color.

**Figure 2:**
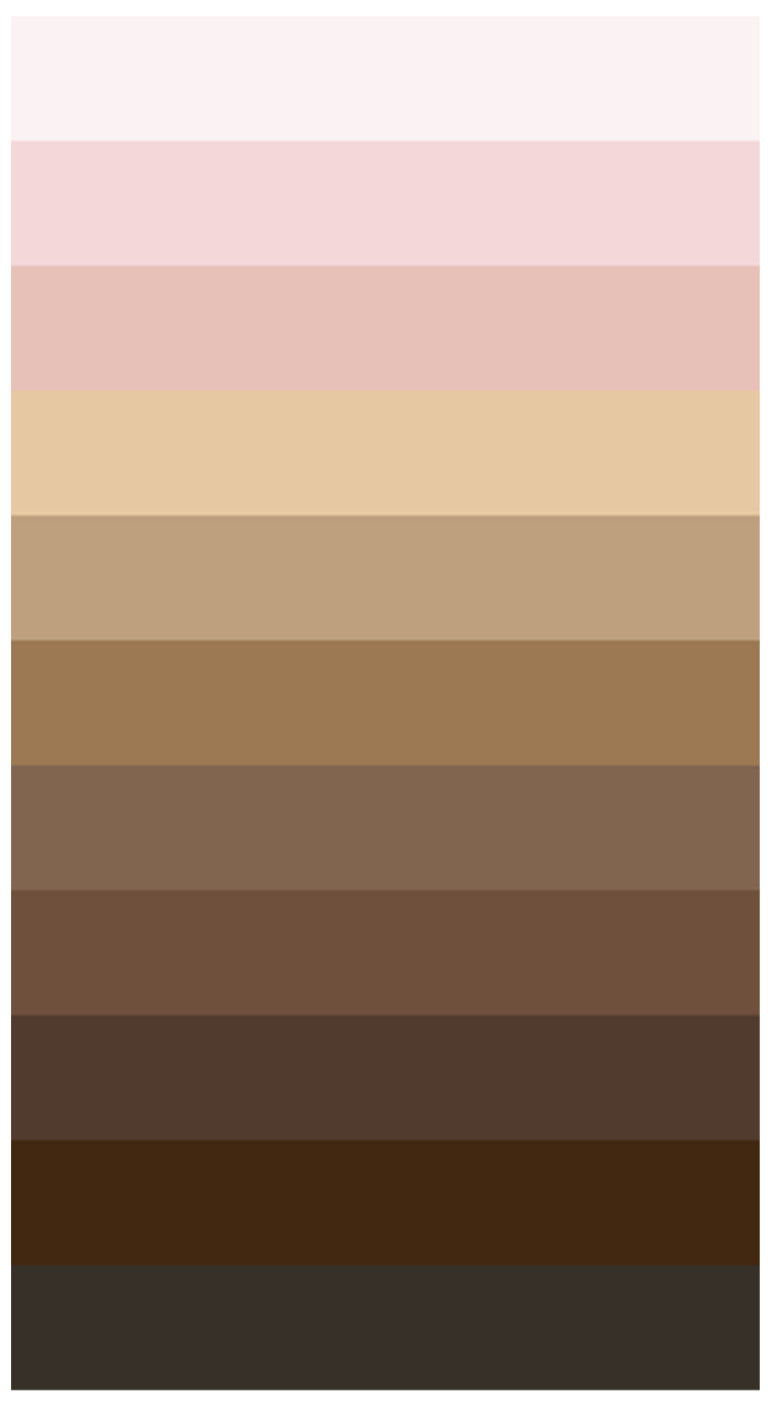
Color palette of possible human skin colors [31].

An 11-color skin color palette was used to represent the range of possible human skin colors, as shown in Figure 2. The skin color palette was validated for international contexts and the full development of the palette is available elsewhere [31]. HEX color values for each portion of the palate are available in Table 1 CIELAB color space values were also extracted from each of the 11 skin colors in the palette. The color of each hearing aid model was compared to all 11 skin colors using the CIELAB ΔE* color-difference formula, an international standard and validated formula. The exact formulas used to perform the comparisons are reported in depth elsewhere [32]. The skin color with the smallest difference value for each hearing aid was recorded as that device’s most similar skin color.

## 3. Results

**Figure 3:**
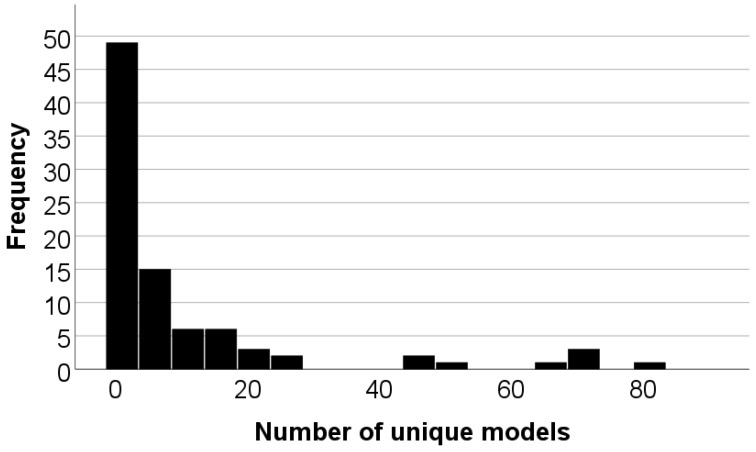
Number of models per listing.

The number of listings in each product code category is shown in Table 2. After duplicate listings were removed, 89 listings were included in the final sample. The listings varied widely in the number of model numbers included in the listings, with most firms registering only 1 device and some firms registering up to 80 unique model numbers, as shown in Figure 3. The countries represented in the sample are shown in Table 3. In total, 997 unique models were listed in the database.

Out of the 997 unique models, images of 236 could be found. A definite match was found between the model number listed in the FDA database and the image listed online for 65.3% (*n* = 154) of these devices. A total of 61 (25.8%) devices were found for companies that did not list a specific model number on their FDA submission. A total of 103 (43.6%) devices that were clearly not typical skin colors (silver, blue, etc.) were excluded from the skin-color analyses. The final sample of hearing aid models that were meant to represent possible skin colors and were listed in the FDA database comprised 83 unique models.

**Figure 4:**
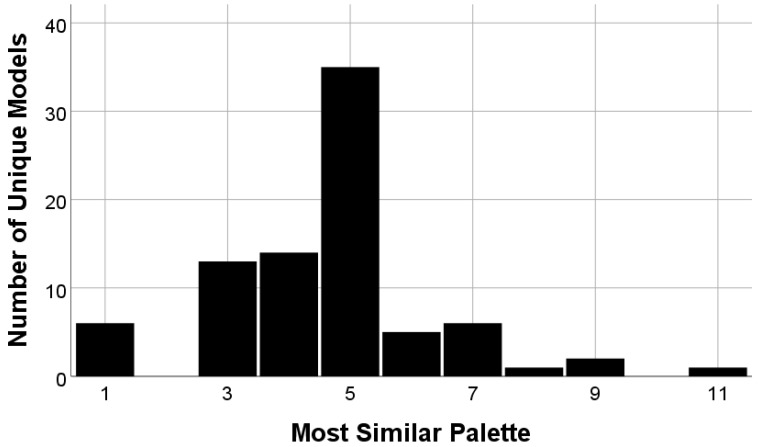
Proportion of hearing aids by skin color.

The proportion of hearing aids most like each skin color palette is shown in Figure 4 and Table 3. The hex color code for each skin color in the palette is also provided. Far more hearing aids in the sample were offered in relatively lighter colors, with few or no options available for darker skin colors. Among skin-color hearing aids that could be conclusively matched to submissions to the FDA database, the five lightest skin colors accounted for 81.9% of devices.

## 4. Discussion

### 4.1. Major Findings

This study has revealed that FDA-cleared OTC hearing aids are 82% more likely to be offered in relatively lighter skin tones aligning with PERLA tones 1–5 vs. relatively darker skin colors (82% vs. 18%, respectively). For the four darkest skin tones, only 4.8% of OTC hearing aids on the market offered a matching product. It is well documented that racism and the priority of product design for lighter skin tones has persisted in Western culture for hundreds of years [33]. This is what informed the initial hypothesis that product offerings would favor lighter skin tones, which, as discovered in Section 3, was determined to be true. This means that BIPOC individuals are much less likely to have access to OTC hearing aids that best reflect their skin tone, which both perpetuates systemic racism in product development and limits more affordable hearing care options due to the stigma that can come with visibly wearing a hearing aid.

### 4.2. Importance of Findings

BIPOC hearing aid users are subject to increased stigmatization due to their intersectional racialized identity and disability [19]. There are several consequences that may result from the OTC color offering inequity. For example, BIPOC people may opt for an in-the-ear hearing aid to minimize device visibility, but this may not be the best product choice for their type of hearing loss [13]. This group of hearing aid wearers is also at an increased risk of bullying and disability-related harassment in addition to the often-daily hate toward their skin color. One of the most devastating implications is that OTCs were intended to be an affordable introduction into receiving support for hearing loss. With BIPOC people already more likely to be living in a lower income bracket, OTCs could have been a better option to help mitigate the consequences of untreated hearing loss, such as dementia and social isolation [11,13].

In noting the disparity between product offerings for different skin tones, further implications arise. There is a history of inequity for BIPOC people attempting to access healthcare in the United States that continues to be pervasive in the present day [33]. Hearing care is a form of healthcare, and it is not immune to the institutional racism existing in all levels of healthcare. This discrimination that is so deeply ingrained in American society has led to a deep mistrust in institutions by BIPOC individuals and can cause these individuals to be more likely to avoid seeking treatment, including visiting an audiologist [13].

Because OTCs are still relatively new, the research from this paper on the skin tone offerings for these devices is unique but warrants further reflection and expansion to other hearing-health-related devices. These findings may allow OTC manufacturers to improve the acceptability of their product offerings for BIPIC people early in the life cycle of the United States OTC market. Hearing aid colorways have also not been traditionally compared to the PERLA skin tone chart, but while this may be novel, the method is still used in many disciplines [31,34]. Additionally, these methods may be valuable in analyzing the availability of prescription hearing aids across the spectrum of human skin colors.

The OTC products that were examined were not put into the context of the company/device’s market share value. This allows for a possible alternative explanation where the diverse brand options take up a larger percentage of the market, meaning that OTC devices would still allow for adequate colorway options for BIPOC individuals in most products available. While this context is important to consider, it does not negate the fact that there are still enough companies offering only lighter skin tone colors as device options that there is a clear skew available products for purchase. BIPOC people have less diversity in brand choices due to racial discrimination, and it just may be less evident in a high-level market analysis because of the market shares of particular devices.

### 4.3. Limitations

The primary limitation is the constraint of finding complete device codes with model colors clearly displayed. Out of 997 unique OTC models registered in the FDA database, only 83 unique OTC hearing aid models were able to be compared according to the study criteria due to incomplete product information in the FDA database. Further studies should incorporate a larger device base, possibly from OTC hearing aid markets in other countries. Future studies should also consider the market share of the individual devices/brands and how colorway offering may impact sales. This will aid in comprehending the intentionality behind the colorways as well as link back to a larger movement occurring in the marketing industry.

## 5. Conclusions

Over-the-counter hearing aids have great potential to improve equity in hearing healthcare for BIPOC individuals. They are cheaper, do not require a prescription from a healthcare professional, and can be a good starting point for people who have mild-to-moderate hearing loss. Based on this project, it has become clear that while these benefits may exist for people with relatively lighter skin, BIPOC people do not have the same opportunities to choose devices that best fit their skin tones. When discretion is a common desire for new hearing aid wearers, the lack of choice offered to BIPOC individuals by the current OTC market is further perpetuation of the racist history plaguing the United States. Future OTC offerings should be expanded to allow for more diverse colorways to afford BIPOC people the same range of product choices that white people are currently allowed.

## Figures and Tables

**Table 1:** Proportion of hearing aids by skin color.

Palette Color	Hex Color Code	Skin Color Only %	Definite Model Match and Skin Color Only %
1	FBF2F3	6.0	7.2
2	F4D7D9	0.8	0
3	E7C1B8	13.5	15.7
4	E6C8A2	21.1	16.9
5	BEA07E	41.4	42.2
6	9C7953	6.8	6.0
7	81654F	6.8	7.2
8	6F503C	0.8	1.2
9	513B2E	1.5	2.4
10	422811	0	0
11	373028	1.5	1.2

**Table 2:** Product codes in FDA database.

Product Code	Description	Number of Entries	Number of Entries, Duplicates Excluded
QUF	Preset hearing aids without wireless capability	45	43
QUG	Preset hearing aids with wireless capability	56	38
QUH	Self-fitting hearing aids with user input for sale online or in physical stores	15	4
QDD	Self-fitting hearing aids with user input for sale online	18	4

**Table 3:** Listings in the database by country of origin.

Country	N	%
Australia	1	1.1
China	55	61.8
Denmark	1	1.1
Mexico	1	1.1
Philippines	1	1.1
South Africa	3	3.4
South Korea	1	1.1
Taiwan	2	2.2
USA	23	25.8
Vietnam	1	1.1

## Data Availability

Data used in this study can be made available by contacting the authors.
